# Antibacterial and antioxidant potential analysis of *Verbascum sinaiticum* leaf extract and its synthesized silver nanoparticles

**DOI:** 10.1016/j.heliyon.2024.e24215

**Published:** 2024-01-11

**Authors:** Jije Mideksa Geyesa, Tarekegn Berhanu Esho, Belete Adefris Legesse, Aselefech Sorsa Wotango

**Affiliations:** a Department of Industrial Chemistry, College of Applied Science, Addis Ababa Science and Technology University, Addis Ababa, Ethiopia; b Center for Innovative Drug Development and Therapeutic Trials for Africa (CDT-Africa), College of Health Sciences, Addis Ababa University, Addis Ababa, Ethiopia; c Center of Excellence for Biotechnology and Bioprocess, Addis Ababa Science and Technology University, Addis Ababa, Ethiopia

**Keywords:** *Verbascum sinaiticum*, Silver nanoparticles, Antibacterial, Antioxidant, Leaf extract

## Abstract

The potential applications of plant extract and nanoparticles in antibacterial and antioxidant studies have garnered significant interest in recent times. Despite being utilized in Ethiopian traditional medicine, *Verbascum sinaiticum* (qetetina) constituents and its usage in nanoparticle synthesis remain relatively unexplored. This study explores the potential of the plant extract and its nanoparticles for antibacterial and antioxidant applications, with a focus on the leaf extracts and its silver nanoparticles. The leaf extract was analyzed using LC-MS and GC-MS and found to contain over 70 compounds, including glycosides, phenolic compounds, flavonoids, and fatty acids. The synthesized nanoparticles had a maximum absorbance of 408 nm, with a size range of 2–40 nm and showed a spherical shape. Using the agar well diffusion method, the extract and nanoparticles were evaluated against Gram-positive (*Staphylococcus aureus* ATCC 2592, *Streptococcus agalactia* ATCC12386) and Gram-negative bacteria (Acinetobacter baumannii ATCC19606, Pseudomonas aeruginosa ATCC27853) bacterial strains. In terms of antibacterial effects, both the silver nanoparticles and leaf extract displayed a greater impact on gram-positive bacterial strains over gram-negative bacterial strains. Additionally, the tests for lowest inhibitory and bactericidal concentration indicated similar outcomes. Notably, the silver nanoparticles exhibited greater antibacterial activity compared to the leaf extract alone. The DPPH (2, 2-diphenylpicrylhydrazyl) assay was conducted to investigate antioxidant activity. The results showed that the plant extract had an IC_50_ value of 143 μg/ml, while the synthesized nanoparticle had an IC_50_ value of 216 μg/ml, indicating that the plant extract had greater antioxidant activity than the synthesized silver nanoparticles.

## 1 Introduction

Plants with medicinal properties or that have a positive pharmacological effect on an organism are known as medicinal plants [1]. These plants' activities are triggered by phytochemicals found in a variety of their parts, including leaves, flowers, seeds, bark, roots, and pulps [2]. Plant phytochemicals are classified as primary or secondary metabolites based on their role in plant metabolism. Primary metabolites include common sugars, amino acids, vitamins, organic acids, and other substances. Flavonoids, tannins, lignin, alkaloids, glycosides, and others are examples of secondary metabolites. These constituents are primarily responsible for the therapeutic activities of medicinal plants in both human and animal species [3]. The presence or absence of various plant constituents is confirmed using phytochemical analysis. It is critical for discovering new therapeutic and industrially useful compounds found in medicinal plants [4]. This category includes two types of analysis: qualitative analysis and quantitative analysis. Quantitative analysis determines the amount or concentration of a phytochemical present in a plant sample, whereas qualitative analysis determines the presence or absence of a phytochemical [5]. In comparison to qualitative analysis, quantitative analysis is a more comprehensive and useful method because the findings can be used for drug discovery, standardization of herbal drugs, explanation of plant medicinal potentials, and plant toxicity levels determination [6].

Plant-mediated synthetic approaches for metal nanoparticle synthesis have emerged to avoid the often-cited drawbacks of conventional synthetic routes, by effectively reducing and stabilizing these materials while making them biocompatible for biological cells [7]. This synthesis method is environmentally friendly, simple, and inexpensive. One of the most important and fascinating types of metallic nanoparticles are silver nanoparticles.

Silver nanoparticles are unique among metallic nanoparticles due to their exceptional physiological properties, which stem from silver's natural antibacterial attributes and its ability to form silver salts through reduction. They also have a large surface area to volume ratio, making them versatile for various applications in fields such as biomedicine, biosensors, catalysis, and pharmaceuticals. Overall, silver nanoparticles are a valuable tool in the world of nanoscience and nanotechnology [7].

The utilization of plant extracts in medicinal practices is well-documented, and recent studies have focused on their potential in synthesizing nanoparticles for enhanced biological activity. Noteworthy research has been conducted in this area. For example, Akintola and his colleagues found that *Blighia sapida* leaf extract has promising antioxidative properties in synthesizing nanoparticles. Similarly, Antonysamy and his coworkers utilized the O*dontosoria chinensis* (L.) plant to synthesize silver nanoparticles, exhibiting potential toxicity, anti-diabetic, and anti-inflammatory effects. Additionally, Corciovă and his team used *Lythrum salicaria* extract to synthesize silver nanoparticles with remarkable antibacterial and antioxidant capabilities [[8], [9], [10]]. These studies highlight the exceptional potential of plant extracts in producing silver nanoparticles with enhanced medicinal properties when compared to the extracts alone. Consequently, plants hold great promise as a source for the synthesis of silver nanoparticles. An example of such a plant is *Verbascum sinaiticum* (qetetina), a biennial plant abundantly available in Ethiopia. The leaves of this plant have long been used in traditional medicine for treating ailments such as stomachaches, viral infections, cancer, sunstroke, fever, and more [11]. Although several studies have been conducted on Verbascum sinaiticum, including its cytotoxic properties and antitrypansomal activity, no detailed investigation has explored the plant's phytoconstituents or its potential for synthesizing silver nanoparticles and evaluating their antioxidant and antibacterial activities [[12], [13], [14], [15]]. Hence, the objective of this study is to identify the constituents present in *Verbascum sinaiticum* and utilize the plant's leaf extract for the production of silver nanoparticles. Additionally, the antibacterial and antioxidant capabilities of both *Verbascum sinaiticum* and its silver nanoparticles are assessed to see any potential improvement in the antibacterial and antioxidant efficacy of leaf extract when used as a precursor for synthesis of silver nanoparticles.

The investigation involved several analyses, including preliminary phytochemical screening, GC-MS, and LC-MS analysis to determine the plant's phytochemicals. Subsequently, the optimal conditions for synthesizing silver nanoparticles using silver nitrate salt are determined. Spectrophotometric methods were employed to characterize synthesized silver nanoparticles, and agar well diffusion method is utilized to assess their antibacterial activity, following the guidelines provided by the Clinical and Laboratory Standards Institute. Additionally, the DPPH free radical scavenging assay is employed to evaluate the antioxidant activity of the leaf extract and its silver nanoparticles.

Through this comprehensive study, valuable insights into the constituents of Verbascum sinaiticum leaf extract, as well antibacterial and antioxidant activities of its synthesized silver nanoparticles can be obtained.

## 2 Methods and materials

### 2.1 Sample preparation

*Verbascum sinaiticum* leaves were harvested in the Yeka region (Addis Ababa). Taxonomists at Addis Ababa University's Department of Plant Biology and Biodiversity Management certified the validity of the plant sample. The plant extract was then prepared in the manner described by Mergia and colleagues with minor modifications [14]. After being repeatedly rinsed with distilled water to remove dirt, soil, and other foreign elements, the collected leaves were spread out on a table and allowed to dry for four weeks in the shade. The dried leaves were pounded into powder using a mortar and pestle. The powdered plant material was then macerated in 1000 ml of methanol for 72 h before being filtered with a Diaphragm Vacuum Pump (Model HS-0153). The filtrate was then dried under reduced pressure in a rotary evaporator (Bucci Rotavapor R-200) and the extract was placed in a refrigerator to be stored at 4 °C after drying and weighing. The percentage yield was calculated by using the formula below in Eq. (1) to check solvent's efficiency.

(1) $$\text{Percentage}\;\text{yield}=\frac{\text{Weight}\;\text{of}\;\text{dry}\;\text{extract}}{\text{Weight}\;\text{of}\;\text{dry}\;\text{plant}\;\text{material}}\times 100$$

### 2.2 Phytochemical screening

Standard chemical methods like Dragendorff's reagent, Alkaline reagent test, Frothing test, Fehling's test, Ferric chloride test, Bontrager's test and Liebermann's test were used to confirm either the absence or presence of alkaloids, flavonoids, saponins, anthraquinones, phenols and Cardiac Glycosides.

### 2.3 Synthesis of silver nanoparticles

Following the procedure by Tamilarasi and Meena with slight modification, silver nanoparticles was synthesized by taking 1: 9 vol ratio of *Verbascum sinaiticum* extract to silver nitrate. The plant extract serves as a reducing and capping agent whereas silver nitrate was used as a precursor [16].

### 2.4 Characterization of silver nanoparticle

The optical property of biosynthesized silver nanoparticles sample was measured at room temperature using a UV–Vis spectrophotometer (JASCO, V-770 spectrophotometer, Germany) between 300 and 800 nm to monitor the completion of bio-reduction of silver ions in aqueous solution.

FTIR (Thermo scientific, iS10 FTIR, USA) spectrometer was used to determine the organic functional groups linked to the surface of silver nanoparticles and the spectra were obtained in the range of 4000–400 cm^−1^. The crystallographic nature of the biosynthesized silver nanoparticles was also determined using an advanced X-ray Diffractometer (Shimadzu XRD-7000, Japan) with Cu–K radiation of wavelength 1.5406 Å and scanning angle 2θ from 10° to 80°. The particle size of the prepared nanoparticle was determined using Scherrer's equation, which is shown in Eq. (2) below.

(2) $$D=\frac{0.9\lambda }{\beta \;\cos\;\theta }$$

where: D is the crystal size, λ is the wavelength of X-ray, Ө is the Braggs angle in radians and β is the full width at half maximum of the peak in radians [17].

The morphology and elemental composition of the silver nanoparticles were analyzed by using SEM-EDX (Hitachi, TM3000, Japan) Whereas the surface charge of the nanoparticles was determined by using Zeta potential instrument (Malvern Zetasizer Nano-ZS ZEN 3600, USA).

By monitoring the weight change that occurs as the sample is heated, a thermo-gravimetric analyzer (AZO materials, TG-209 F3 Nevio, UK) was used to determine a material's thermal stability and percentage weight loss. In addition, Transmission electron microscopy (JEOL JEM-2100 LaB6, Japan) was used to determine the shape and size distribution of the nanoparticles formed.

### 2.5 Antibacterial and antioxidant activities

The antimicrobial activity of both the plant extract and the silver nanoparticles were determined using the agar well diffusion method following the procedure by Jafari-Sales and colleagues with slight modification [18]. Four pathogenic bacterial strains, two Gram-positive (*Staphylococcus aureus* ATCC 2592, *Streptococcus agalactie*) and two Gram-negative (*Acinetobacter baumannii* ATCC19606, *Pseudomonas aeruginosa* ATCC 27853) were used. These bacterial strains were obtained from Center for Innovative Drug Development and Therapeutic Trials for Africa (CDT-Africa) laboratory, Addis Ababa University. The antioxidant test procedure was adapted from Xiao and coworkers, the antioxidant activity of the plant extract and silver nanoparticles were determined using 0.1 mM DPPH (2,2-diphenyl-1-picrylhydrazyl) solution and ascorbic acid as a standard [19].

## 3 Results and discussion

### 3.1 Phytochemical analysis

**Fig. 1:**
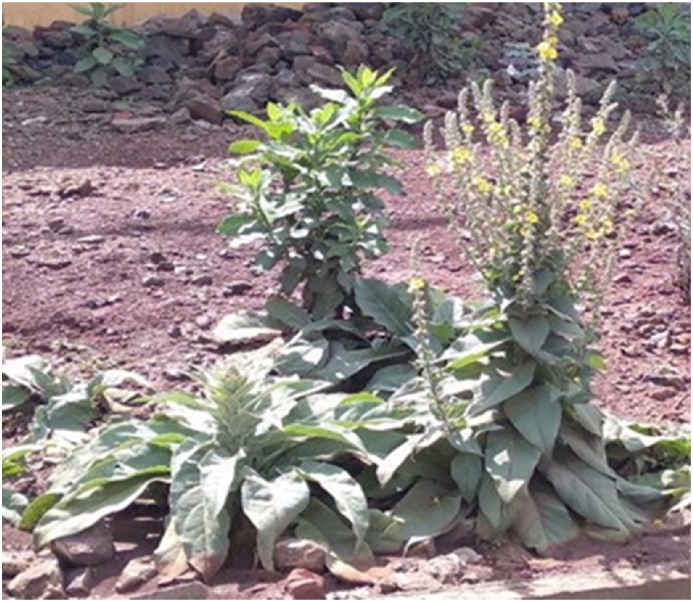
The plant picture.

Mergia and colleagues discovered percentage yield of *Verbscum siniaticum* be 18.13 %, whereas this study discovered an 18.4 % percentage yield. This demonstrates that the discovered result is consistent with previous report on the plant [12,14]. The picture of the plant is shown in Fig. 1.

Phytochemical screening, which involved color changes, precipitate formation, and revealed the presence of useful bioactive compounds such as alkaloids, tannins, flavonoids, terpenoids, phenols, saponins, and anthraquinones in leaf extracts. The color changes can be seen from Fig. S1 and the results for present/absence of phytochemicals are shown in Table 1.

**Table 1:** Phytochemical screening result of *Verbascum sinaiticum* leaf extract.

S/No	Bioactive phytochemicals	Test/Reagents	Results
1	Alkaloids	Dragendoff's test	+
2	Flavonoids	Alkaline reagent	+
3	Saponins	Frothing test	–
4	Carbohydrates	Fehling's test	+
5	Phenols	Ferric chloride test	+
6	Anthraquinones	Bontrager's test	–
7	Cardiac Glycosides	Liebermann's test	+

**Note:** + = Present, and - = Absent.

As shown in Table 1, the bioactive compounds like alkaloids, flavonoids, carbohydrates, phenols and cardiac glycosides were present whereas anthraquinone and saponins were absent in methanolic leaf extract of *Verbascum sinaiticum*. Mergia and colleagues [14] report backs up this finding. The carbohydrate test and cardiac glycoside test, on the other hand, are new assays performed for the first time on this plant and yield positive results. This shows that this plant includes glycosides and sugars in addition to the phytochemicals studied. This was supported by LC-MS and GC-MS analyses, which revealed a variety of carbohydrates and glycosides in *Verbascum siniaticum* leaf extract. Furthermore, the phytoconstituents that are present are responsible for the bio reduction of Ag + to Ag0 as well as the stability of V-AgNPs [20].

**Fig. 2:**
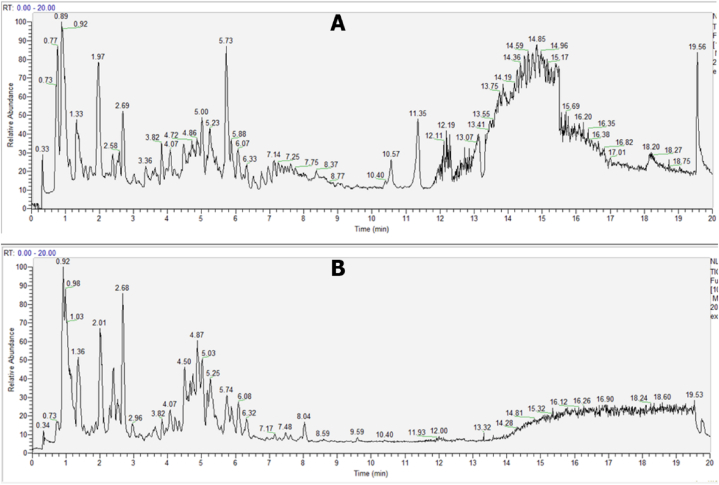
LC-MS total ion chromatogram of *Verbascum sinaiticum* (A) Total ion chromatogram of plant extract in positive mode, (B) Total ion chromatogram of plant extract in negative mode.

LC-MS analysis of methanol extracts of *Verbascum sinaiticum* had detected several peaks with different retention time. By using natural chemicals, natural products, and Chinese medicine as references from the database, compound discover software was able to identify the compounds present in the plant extract which are listed in Table S1. The peaks are illustrated in Fig. 2.

The peaks discovered had varying strengths, and the identified compound name, molecular formula, retention duration, and compound class were determined and listed in Table S1. Six of the identified compounds listed in the table have been reported to have high medicinal value when compared to the rest of the compounds found in the plant extract. Verbascoside A, Scropheanoside I, Picroside II, Phlinoside A, Harpagoside, and Geniposidic acid are the compounds names. These compounds have antioxidant, anti-inflammatory, anticancer, and antiviral properties [[21], [22], [23], [24]]. Verbascoside and Scropheanoside I are two phenylpropanoid compounds with the same molecular formula (C_31_H_40_O_16_), but they exhibit different retention times on analysis, with Verbascoside having a retention time of 6.0 min and Scropheanoside I having a retention time of 6.319 min. This difference in retention time suggests the possibility of structural variations or isomeric forms between the two compounds. Picroside II, on the other hand, is a tannin compound with a molecular formula of C_23_H_28_O_13_ and a retention time of 4.24 min. Its identification as a tannin highlights the presence of secondary metabolites in the sample, which may have potential biological activities. Phlinoside A, another phenylpropanoid compound, stands out with its unique profile among the detected compounds. It has a molecular formula of C_35_H_46_O_20_ and a retention time of 4.149 min, indicating distinctive structural characteristics and potential bioactive properties. N-Acetyl-L-leucine, with a molecular formula of C_8_H_15_NO_3_ and a retention time of 4.297 min, is identified as an alpha amino acid. Its presence suggests that Verbascum sinaiticum contains essential building blocks for protein synthesis. Additionally, L-Glutathione oxidized, a disulfide compound with a molecular formula of C_20_H_32_N_6_O_12_S_2_ and a retention time of 1.367 min, is detected in the plant extract, indicating the potential involvement of oxidative stress pathways. These examples illustrate the variations in retention times or fragmentation patterns observed in the compounds detected during LC- MS analysis of Verbascum sinaiticum extract. Furthermore, the compounds found can be predicted to have reducing and stabilizing effect during the synthesis of silver nanoparticles.

GC-MS was used to identify bioactive volatile compounds in leaf extract of *Verbascum sinaiticum.* The compounds were identified using mass spectrometry in conjunction with GC. The various components detected by GC-MS in the leaf extract are listed in Table S2 and the total ion chromatogram spectra can be seen in Fig. S2. The constituents present in the plant are numerous and can be expected to have a variety of benefits including a reduction and stabilization effect during the synthesis of silver nanoparticles [7].

### 3.2 Silver nanoparticles synthesis and characterization

**Fig. 3:**
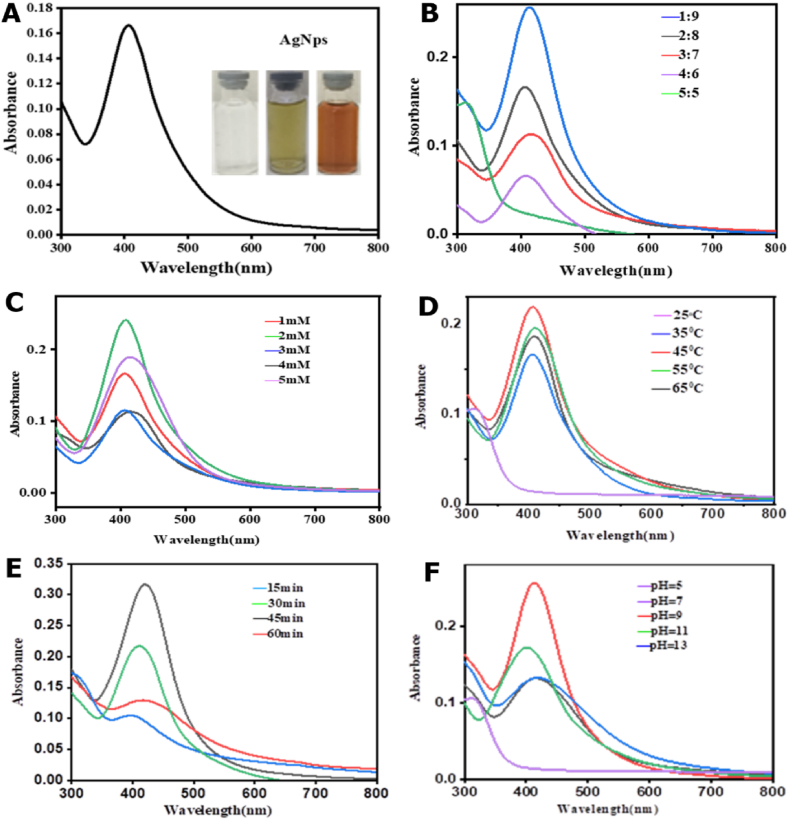
Effects of (A) Maximum absorption and visual confirmation of silver nanoparticles formed, (B) ratio of plant extract to silver nitrate, (C) silver nitrate concentration, (D) Temperature, (E) stirring time, (F) pH optimization.

Silver nanoparticles were successfully synthesized using silver nitrate solution and *Verbascum sinaiticum* leaf extract under optimum conditions. The formation of silver nanoparticles was indicated by the color change from yellow to dark brown as shown in Fig. 3(A). Color transitions occur due to molecular and structural changes, in the test substances leading to corresponding changes in the ability to absorb light in the visible region of the electromagnetic spectrum [[25], [26], [27]

The UV–Vis spectra revealed a strong absorbance at 408 nm, indicating the formation of silver nanoparticles. This is shown in Fig. 3(A). The finding is very close to the majority of results published in various journals by Lomeli-Rosales and coworkers, Tamilarasi and Meena, and Hemlata and coworkers [16,20,25]. This result strongly agrees with the previous range of maximum wavelength values of silver nanoparticles that range from 400 to 450 nm of other medicinal plants besides *Verbascum sinaiticum*.

The synthesis of silver nanoparticles is heavily reliant on a few operational parameters. These parameters have an impact on nanoparticle synthesis regardless of the technique used. Several important experimental factors were evaluated in this study, including stirring time, silver nitrate concentration, leaf extract to silver nitrate ratio, temperature, and pH. The most effective parameters were determined and used in bulk preparations [26].

The concentration ratio of leaf extract to silver nitrate was optimized by increasing the concentration of leaf extract (1, 2, 3, 4, and 5 ml) in 10 ml total solution [27]. Silver nitrate added were (9, 8, 7, 6, 5 ml). The proportions were 1: 9, 2: 8. 7: 3, 6: 4 and 5: 5. From the ratio studied 1:9 was found to have the highest absorbance, indicating the highest amount of silver nanoparticle formation when compared to the others. This is shown in Fig. 3(B). The result found is consistent with other reports done on using *Odontosoria chinensis* (L.) extract by Antonysamy Johnson and coworkers [9].

The absorption peak of the reaction mixture changed with silver nitrate concentrations ranging from 1 mM to 5 mM. UV-graph showed that 2 mM more absorbance than the other concentrations which is confirmed by the intensity of the peaks obtained in the graph. The result confirmed that the optimum value of the concentration of the silver nitrate is 2 mM, which was used for further investigations Fig. 3(C) illustrates this.

The effect of reaction time was studied by monitoring the reaction of plant extract and silver nitrate solution for 15, 30, 45, and 60 min, by keeping other optimal conditions constant. The absorbance value increases over time, peaking in 45 min. From 45 to 60 min, the absorbance value begins to fall. This is shown in Fig. 3(E). Furthermore, nanoparticle agglomeration was observed as the color of the synthesized nanoparticles changed from brown to dark brown indicating the formation of unstable and large particles. As a result, the optimal stirring time for the formation of silver nanoparticles from *Verbascum sinaiticum* leaf extract was determined to be 45 min in this study. This finding is consistent with previous researches done on other biosynthesized silver nanoparticles using *Piper retrofractum* Vahl fruit extract and potato (*Solanum tuberosum*) infusion by Amaliyah and colleagues, Roy and his coworkers [27,28].

The effect of temperature was investigated by varying the temperatures to 25 °C, 35 °C, 45 °C, 55 °C, and 65 °C while maintaining other optimal conditions constant. Fig. 3(D) shows the absorbance increases with increasing temperature until it reaches 45 °C. Between 55 °C and 65 °C, absorbance decreased while aggregation increased. As a result, 45 °C was chosen as the optimal temperature. Others reported that the optimal temperature for the synthesis of silver nanoparticles from various plant extracts was 25–80 °C [29,30]. This result is consistent with previous works using different plant to synthesize silver nanoparticles by Tamilarasi and Meena who have used *Gomphrena globosa* (Globe amaranth) leaf extract for the synthesis of silver nanoparticles [16].

pH effect on the synthesis of silver nanoparticles was investigated. Three different pH conditions were examined: acidic, neutral, and basic, with pH values varying from 5, 7, 9, 11, and 13. The results, shown in Fig. 3(F), revealed that when the pH was 5 (acidic), the UV–Vis spectra did not exhibit the surface plasmon resonance peak. However, as the pH was increased to 7, the peak started to appear, indicating the initiation of silver nanoparticle synthesis. The maximum intensity of the absorbance peak was observed at a pH of 9, suggesting that the highest biogenic synthesis of silver nanoparticles occurred under alkaline conditions. This result is consistent with the results obtained when optimizing silver nanoparticles from Olive and green tea leaf extracts and *Ginkgo biloba* leaf, which have an optimum pH of 9–11 by Bergal and coworkers and Huang and his colleagues [30,31].

**Fig. 4:**
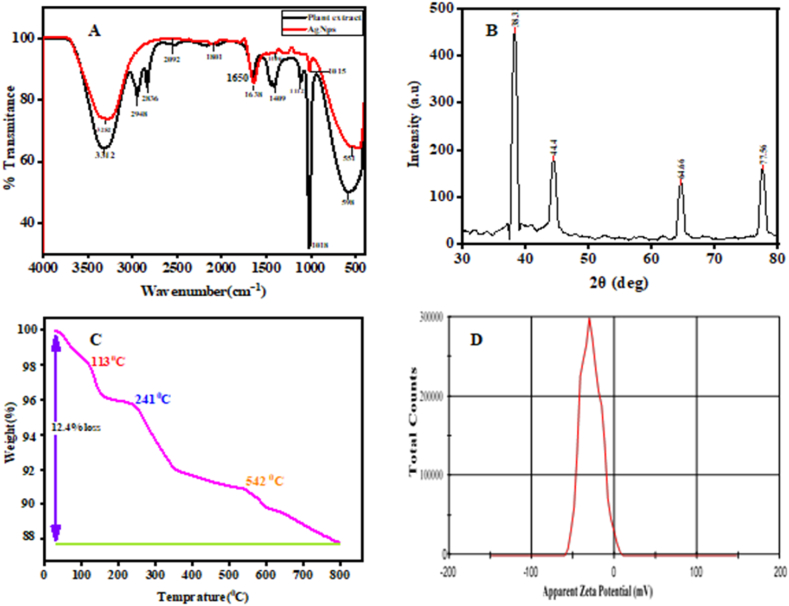
Characterization of silver nanoparticles by (A) FTIR, (B) XRD, (C) TGA, (D) Zeta potential.

FTIR study was conducted to confirm the functional groups (which are responsible for reduction, stabilization, and capping agents) in order to verify the capped biomolecules with silver nanoparticles [10]. It was then compared with the FTIR spectrum of leaves extract. The bands shown in Fig. 4(A) shows FTIR spectra of silver nanoparticles corresponds to 3282 Cm^-1^ (O–H, Alcohol), 2092 Cm^-1^(N=C=S stretch, isothiocyanate), 1801 Cm^-1^(C=O stretch, conjugated acid halide), 1638 Cm^-1^(C=C, stretch, conjugated, alkene), 1406 Cm^-1^(C=C, bend, aromatic compounds), 1015 Cm^-1^ (C–F stretch, alkyl halides) and 551Cm^-1^ (C–I, Halogen Compounds) which signify the presence of phytoconstituents reduction during synthesis [28]. The spectra of plant extract correspond to different functional groups and class of compounds which includes 3312 Cm^-1^ (O–H stretch, alcohol), 2948 and 2836 Cm^-1^(C–H stretch, alkane), 1650 Cm^-1^(C=C stretching, Conjugated alkene), 1409 Cm^-1^(O–H bending, Carboxylic acid), 1112 Cm^-1^(C–N stretch, amine), 1018 Cm^-1^ (Ethers, Alcohols and sugars) and 598 Cm^-1^(C–Cl, Halogen compound), respectively. The intensity and position of the formed peaks changed after silver nanoparticle synthesis. The majority of the peaks correspond to polyphenolic groups, triterpenoids, alkaloids, steroids, and tannins, which are abundant in the leaf extract and aid in the formation of silver nanoparticles [22]. This finding is consistent with other greenly synthesized silver nanoparticles and their leaf extracts by Amin and coworkers and Lomeli and his colleagues who have used leaf extract of *Capsicum chinense* plant and *Solanum xanthocarpum* L. berry extract to synthesize silver nanoparticles [25,32].

The X-ray diffraction (XRD) pattern of the biosynthesized silver nanoparticles shows four diffraction peaks at 2θ = 38.32, 44.4, 64.66 and 77.56° in Fig. 4(B) which are corresponding sets of lattice planes to the (111), (200), (220) and (311) were observed. They are indexed as the band for face centered cubic structures of silver. The XRD pattern shows that the formed silver nanoparticles are crystalline in nature [33]. According to the XRD patterns, the highest peak intensity of (111) plane with narrow full width at half maximum (FWHM) demonstrates the good crystalline nature of synthesized silver nanoparticles. The resulting peaks and Bragg's reflections are strongly agreed upon by the Joint Committee on Powder Diffraction Standards (JCPDS, file no. 04–0783). Using Debye-equation, Scherrer's the average crystallite sizes of the particles were calculated (Equation (2)). It was discovered that the synthesized silver nanoparticles had an average crystalline size of 14 nm. The sharp Bragg peaks could have been caused by a capping agent that stabilized the nanoparticle. Intense Bragg reflections indicate the presence of strong X-ray scattering centers in the crystalline phase, which could also be caused by capping agents. The process of centrifugation and redispersion of the pellet in Millipore water after nanoparticle formation as part of the purification process ruled out independent crystallization of the capping agents. As a result, the XRD results suggested that the bio-organic phase crystallizes on the surface of the silver nanoparticles or vice versa. The broadening of peaks in solid XRD patterns is generally attributed to particle size effects. This study is consistent with previous works done on other plants by Khodadadi and coworkers and Urnukhsaikhan and his colleagues who used using *Vaccinium arctostaphlyos* extract and *Carduus crispus* extract to synthesize silver naoparticles [34,35].

TGA analysis gives us information on the percent weight loss of silver nanoparticles as temperature rises. Silver nanoparticles showed multi stage decomposition at 113 °C, 241 °C, 542 °C and remains constant until 800 °C in Fig. 4(C). The percentage weight loss was found to be 12.4 % due to the evaporation of water and organic components. Water desorption from the organic component of the NPs causes weight loss in the temperature 0 °C–100 °C. This, in turn, demonstrates that biosynthesized Ag NPs are an excellent candidate for absorbing moisture in a wide range of applications. This study is in line with findings on silver nanoparticles made from Fritillaria flower extract using green synthesis by Hemmati and coworkers [36].

The zeta potential of biosynthesized AgNPs was measured and the results show that the surfaces of the nanoparticles are negatively charged and well distributed in the medium. Particles in a stable suspension have zeta potential values ranging between +30 and −30 mV shown in Fig. 4(D). This study found −28.1 mV zetapotential value which indicates the silver nanoparticles synthesized have negative surface charges. This is helpful in electrostatic stabilization against aggregation. The result found is close with other reports done on greenly synthesized silver nanoparticles using *Thunbergia grandiflora* by Thivaharan Varadavenkatesan and coworkers, who have found the zeta size of −24.5 mV [33].

**Fig. 5:**
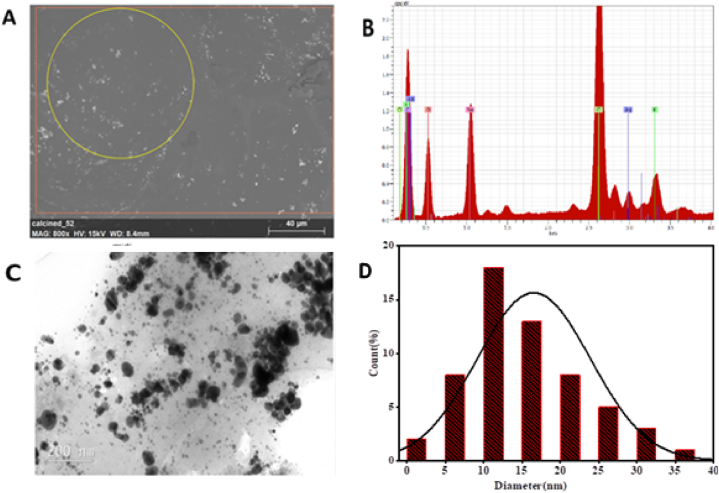
Characterization by (A) SEM, (B) EDX, (C) TEM, (D) Size distribution in (nm).

SEM was used to examine the morphology of the green-synthesized silver nanoparticles. The polymorphic shapes of the prepared nanoparticles some of which were ellipsoidal and irregularly granulated is shown in Fig. 5(A). The elemental analysis revealed elemental composition which includes carbon, oxygen, sodium, potassium, silver and chlorine. This is illustrated in Fig. 5(B). The elements in the sample are linked to the leaf extract's on the surface of the silver nanoparticles and are crucial for the stability and reduction of biosynthesized silver nanoparticles. The reason behind the polymorphic shapes of nanoparticles lies in the thermodynamic and kinetic aspects of the synthesis process. The precursor materials undergo rearrangements and transformations during nucleation and growth, resulting in the development of various crystal forms and surface energies. These innate tendencies, together with the effect of external factors such as surfactants and reaction conditions, contribute to the formation of a wide range of nanoparticle morphologies. Similar results were observed in the biosynthesis of silver nanoparticles using *Cucumis prophetarum* by Hemlata and coworkers [20].

TEM image in Fig. 5(C) demonstrates the spherical nature of the silver nanoparticles. The image supports the findings from the XRD by displaying clusters of small grains and some dispersed nanoparticles. Different mechanisms of nucleation and growth, which are affected by a variety of factors such as temperature, time, pH, and concentration, can explain the differences in morphology and size distribution. The biosynthesized Ag NPs are surrounded by shaded layers of foreign matter, which most likely represent the capping agent from the leaf extract. Image J version 1.8 software was used to determine the size distribution of the silver nanoparticles, which was found to be in the range of 2 nm–37 nm. The silver nanoparticle synthesized in this study was similar to other reports done on different plant by Amaliyah and coworkers, Bergal and his colleagues [28,30].

### 3.3 Antibacterial and antioxidant activity

#### 3.3.1 Antibacterial study

Antibacterial study was done using agar well diffusion method. In agar well diffusion, two antibiotic disks were used as positive controls, Vancomycin (30 μg/disk) and Ciprofloxacin (5 μg/disk), and DMSO as negative control. The type of antibiotic disk used is determined based on the bacterial strain. Vancomycin (30 μg/disk) is used for gram positive bacteria such as *Staphylococcus aureus* and *Streptococcus agalactiae*, whereas Ciprofloxacin (5 μg/disk) is used for gram negative bacteria such as *Acinetobacter baumannii* and *Pseudomonas aeruginosa*.

Fig. S4 and Table 3 illustrated the zone inhibition obtained from plant extract and silver nanoparticles at concentrations of 0.5 mg/ml, 1 mg/ml, 1.5 mg/ml, and 2 mg/ml. It was discovered that the plant extract has less antibacterial activity against the test bacteria than silver nanoparticles synthesized from the plant extract.

**Table 2:** Antibacterial activity of plant extract, silver nanoparticles and silver nitrate measured as diameter (mm) of zone of inhibition.

	Zone of inhibition (mm)									
Bacterial strain	Plant extract (mg/ml)				Silver Nanoparticles (mg/ml) Antibiotic discs					
	0.5	1	1.5	2	0.5	1	1.5	2	Ciprofloxacin 5 μg/ml	Vancomycin 30 μg/ml
									34.9 ± 0.0 21.2 ± 0.0 35 ± 0.0 35.2 ± 0.0	23 ± 0.0 21.5 ± 0.0 23 ± 0.0 22.9 ± 0.0
***P.ae.***	13.8 ± 0.01	14.3 ± 0.01	14.8 ± 0.86	15.6 ± 0.02	15.1 ± 0.06	16.3 ± 0.01	16.8 ± 0.03	17.5 ± 0.03		
***A.ba.***	14.1 ± 0.01	14.2 ± 0.01	14.5 ± 0.02	15.2 ± 0.01	14.3 ± 0.01	15.1 ± 0.01	15.3 ± 0.01	16.2 ± 0.06		
***S. au.***	15.1 ± 0.03	16.2 ± 0.01	17.1 ± 0.01	21.2 ± 0.06	19.1 ± 0.02	21.1 ± 0.06	26.2 ± 0.01	35.1 ± 0.12		
***S. ag.***	13.4 ± 0.06	14.9 ± 0.1	15.1 ± 0.1	17.1 ± 0.03	18.1 ± 0.17	24.3 ± 0.15	31.2 ± 0.15	34.3 ± 0.15		
		**Silver Nitrate (mg/ml)**			M±SD: Mean plus standard deviation, n = 3					
	**0.5**	**1**	**1.5**	**2**						
*P.ae.*	7.2 ± 0.37	8.9 ± 0.17	10.3 ± 0.2	12.9 ± 0.17						
*A.ba.*	8.6 ± 0.18	10.9 ± 0.17	12.2 ± 0.15	14.7 ± 0.5						
*S.au.*	9.0 ± 0.35	10.2 ± 0.68	11.6 ± 0.76	12.7 ± 0.45						
*S.ag.*	8.8 ± 0.52	11.2 ± 0.1	13.3 ± 0.7	14.8 ± 0.14

**Table 3:** MIC and MBC values of plant extract and silver nanoparticles.

Bacterial strain	Plant extract		Silver nanoparticles	
	MIC (mg/ml)	MBC (mg/ml)	MIC (mg/ml)	MBC (mg/ml)
*Pseudomonas aeruginosa*	2	0.5	1	0.5
*Acinetobacter baumannii*	1.5	0.25	1	0.25
*Staphylococcus aureus*	1	0.125	0.5	0.125
*Streptococcus agalactiae*	0.5	0.125	0.25	0.125

MIC = Minimum Inhibitory Concentration, MBC = Minimum Bactericidal Concentration.

At the highest concentration of 2 mg/ml, the plant extract displayed a zone of inhibition of 21.2 mm. In comparison, the silver nanoparticles exhibited a larger zone of inhibition of 35.1 mm against *Staphylococcus aureus*. Against *Streptococcus agalactiae*, the plant extract had a zone of inhibition of 17.1 mm, while the silver nanoparticles achieved a larger zone of inhibition of 34.3 mm at the same concentration. Furthermore, *Pseudomonas aeruginosa* showed a zone of inhibition of 15.6 mm for the plant extract and 17.5 mm for the silver nanoparticles. When tested against *Acinetobacter baumannii*, the plant extract exhibited a zone of inhibition of 15.2 mm, whereas the silver nanoparticles yielded a zone of inhibition of 16.2 mm at the maximum tested concentration. Whereas silver nitrate concentration of 2 mg showed the zone of inhibition for *Acinetobacter baumannii (A. ba.)* is 14.7 mm, for *Pseudomonas aeruginosa (P.ae.)* is 12.9 mm, for *Streptococcus agalactiae (S. ag.)* is 14.8 mm, and for *Staphylococcus aureus (S. au.)* is 12.7 mm. This is also shown in Table 2. When the silver nitrate data are compared to the zone of inhibition found with silver nanoparticles and plant extract, it is clear that silver nanoparticles and plant extract both exhibit higher zones of inhibition than silver nitrate alone. However, the silver nanoparticles containing the combination of the plant extract and the silver nitrate showed the highest zone of inhibitions and this points to a synergistic effect in which the combination of silver nitrate and plant extract improves antibacterial activity beyond what individuals can achieve.

The minimum inhibitory concentration is determined using 96 microwell plate which is demonstrated in Fig. S5. On each bacterial strain tested, the MIC values for both plant extract and nanoparticles are different. The DMSO in the 10th well is used as a dissolving agent for the plant extract. The 11th and 12th wells control growth and sterility, respectively.

MIC was defined as the sample concentration that inhibited microbial growth and stopped the medium from changing color. INT (Iodo-Nitro-Tetrazolium) is an indicator dye that is colorless when oxidized and pink when reduced. When microorganisms metabolize carbon compounds, they produce waste products that act as reducing agents, also known as reductants or electron donors, and turn tetrazolium pink. This is shown in Fig. S5.

The MBC was thought to contain the smallest amount of extract after being removed from the well, spread out on a plate, and incubated for 24 h at 37 °C shows no bacterial growth. This is tabulated in Table 3.

Both the plant extract and the silver nanoparticle have higher antibacterial activity against gram-positive bacteria than gram-negative bacteria, according to this discovery. Furthermore, silver nanoparticles synthesized from the plant extract outperform the plant extract alone in terms of antimicrobial activity. This finding is consistent with previous research on the antimicrobial properties of greenly synthesized silver nanoparticles by Khorrami and coworkers, Pandey and coworkers [37,38].

This variation in activity between gram-positive and gram-negative bacteria cell walls can be linked to structural differences. Antimicrobial substances primarily target Gram-positive bacteria's strong peptidoglycan coating in their cell walls. Because of their potential to break the peptidoglycan layer, the plant extract and silver nanoparticles are most likely effective against gram-positive bacteria. Gram-negative bacteria, on the other hand, have an additional outer membrane that serves as an additional protective barrier. This lipopolysaccharide-based outer barrier can impede the accessibility and efficiency of antibacterial medicines. As a result, the plant extract and silver nanoparticles may be less effective against gram-negative bacteria than they are against gram-positive bacteria. However, silver nanoparticles produced from plant extract have much more antibacterial activity. The unique features of silver nanoparticles, particularly their high surface area-to-volume ratio, can be linked to this increase in activity [39]. The expanded surface area allows for more contact with bacterial cells, improving antimicrobial agent efficiency. Furthermore, silver nanoparticles have natural antibacterial capabilities. They have the ability to emit silver ions, which are poisonous to bacteria because they disrupt biological processes and damage vital components. The combination of the qualities of the silver nanoparticles and the plant extract resulted in a more effective antibacterial impact against both gram-positive and gram-negative bacteria [40].

#### 3.3.2 Antioxidant activity

The (DPPH) 2, 2-diphenylpicrylhydrazyl free radical assay was used to measure the antioxidant activity of the synthesized silver nanoparticles, with ascorbic acid serving as standard. The color will change as DPPH is reduced. When reduced, the color of DPPH changes from purple to yellow.

**Fig. 6:**
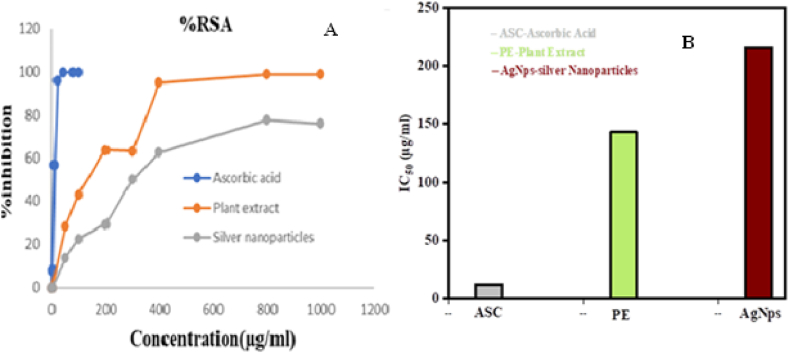
(A) DPPH inhibition of ascorbic acid, plant extract and silver nanoparticles, (B) Bar graph comparing IC_50_ values of ascorbic acid, silver nanoparticles and plant extract.

Fig. 6(A) show the inhibition of ascorbic acid from 2.5 μg/ml to 100 μg/ml concentrations and plant extract and silver nanoparticles inhibition at concentrations ranging from 50 μg/ml to 1000 μg/ml, Fig. 6(B) shows the IC 50 values of ascorbic acid, silver nanoparticles and plant extract which was determined by plotting linear points on a graph and solving the linear equation Y = Mx + b. Ascorbic acid 12 μg/ml, silver nanoparticles 216 μg/ml, and plant extract 143 μg/ml IC50 values were discovered. The IC_50_ value was determined by plotting linear points on a graph and solving the linear equation Y = Mx + b. Ascorbic acid 12 μg/ml, silver nanoparticles 216 μg/ml, and plant extract 143 μg/ml IC_50_ values were discovered. The lower the IC_50_ value, the higher the antioxidant activity. This study found that the antioxidant activity of the leaf extract is higher than that of its silver based nanoparticles. Our findings are unlike other authors who have reported no significant changes in antioxidant potential of leaf extract at the end of the green synthesis [25]. This might be due to variety of reasons, including a decrease in bioactive chemicals, changes in chemical structure, interaction with silver nanoparticles, and silver incompatibility. Various chemical processes occur during the manufacturing of silver nanoparticles, which may result in the breakdown or change of the bioactive molecules responsible for antioxidant activity. As a result, the quantity of these chemicals in silver nanoparticles may be lowered, resulting in diminished antioxidant action. The antioxidant and antibacterial activities test results of silver nitrate can be seen from the supplementary material (Figs. S6 and S7).

## 4 Conclusion

This study found that *Verbascum sinaiticum* contains over 70 non-volatile and volatile compounds. Furthermore, we have found that *Verbascum sinaiticum* is an excellent plant for the synthesis of silver nanoparticles with potent antibacterial properties. The silver nanoparticles synthesized had a maximum absorption wavelength of 408 nm, an average crystalline size of 14 nm, a surface charge of −28 mv, and a spherical shape. The antibacterial activity of both the plant extract and the synthesized silver nanoparticles against gram positive bacteria (*Streptococcus agalactiae, Staphylococcus aureus*) had 21.1 mm and 17.1 mm zone of inhibition for plant extract and 34.3 mm and 35.1 mm for the synthesized silver nanoparticles at 2 mg/ml concentration, respectively. In addition, gram negative bacteria (*Pseudomonas aeruginosa*, *Acinetobacter baumannii*) zone of inhibition was 15.6 mm and 15.2 mm for plant extract and 17.5 mm and 16.2 mm for synthesized silver nanoparticles at 2 mg/ml concentration. The MIC and MBC tests showed silver nanoparticles were effective at lower concentration of 0.25 mg/ml to 1 mg/ml than the plant extract which were effective from 1 mg/ml to 2 mg/ml concentration. The result indicated the synthesized silver nanoparticles exhibited higher antibacterial activity as compared to the plant extract and both have higher antibacterial activity against gram positive bacteria as compared to gram negative bacteria. The antioxidant study reveals that *Verbascum sinaiticum* had higher antioxidant activity with IC_50_ value of 143 μg/ml than the synthesized silver nanoparticles which had IC_50_ value of 216 μg/ml. This medicinal plant needs standardization to be used as a traditional medicine in a scientific way. It can serve as a precursor to synthesize quality silver nanoparticles. It should be noted that the assessment of antioxidant activity was limited to in vitro DPPH assay, further in vitro and vivo antioxidant studies are necessary to validate the potential benefits in a complex biological system. Although prior toxicity studies on the plant extract by Merga and co-workers [14] have demonstrated no observed toxicity effects in Swiss albino mice, it is essential to conduct toxicity studies specific to the formulated nano silver product to ensure the safety of its application in different biological systems.

## Data availability statement

The soft and hard copies of the data are available at Addis Ababa Science and Technology University Library.

## CRediT authorship contribution statement

**Jije Mideksa Geyesa:** Writing - review & editing, Writing - original draft, Methodology, Investigation, Formal analysis. **Tarekegn Berhanu Esho:** Supervision, Resources, Methodology, Investigation, Conceptualization. **Belete Adefris Legesse:** Resources, Methodology, Formal analysis, Data curation. **Aselefech Sorsa Wotango:** Writing - review & editing, Writing - original draft, Supervision, Resources, Methodology, Formal analysis, Conceptualization.

## Declaration of competing interest

The authors declare the following financial interests/personal relationships which may be considered as potential competing interests:Jije Mideksa Geyesa reports equipment, drugs, or supplies was provided by Stockholm University, Sweden. Aselefech Sorsa reports a relationship with Addis Ababa Science and Technology University that includes: employment.
