# Supplementary material for: Antibacterial and antioxidant potential analysis of Verbascum sinaiticum leaf extract and its synthesized silver nanoparticles
Source: Heliyon. 2024 Jan 11;10(2):e24215. doi: 10.1016/j.heliyon.2024.e24215 (PMC10803913; doi:10.1016/j.heliyon.2024.e24215)
Supplement: Multimedia component 1 [file mmc1.docx]

**Antibacterial and antioxidant potential analysis of *Verbascum sinaiticum* leaf extract and its synthesized silver nanoparticles**

Jije Mideksa Geyesa, ^1^ Tarekegn Berhanu Esho, ^1, 3^ Belete Adefris Legesse, ^2^ Aselefech Sorsa Wotango^1, 3^ *

^1^Department of Industrial Chemistry, College of Applied Science, Addis Ababa Science and Technology University, Addis Ababa, Ethiopia.

^2^Center for Innovative Drug Development and Therapeutic Trials for Africa (CDT-Africa), College of Health Sciences, Addis Ababa University, Addis Ababa, Ethiopia.

^3^Center of Excellence for Biotechnology and Bioprocess*,* Addis Ababa Science and Technology University, Addis Ababa, Ethiopia.

*Corresponding Author Email: [aselefech.sorsa@aastu.edu.et](mailto:aselefech.sorsa@aastu.edu.et)

**Experimental section**

**Chemicals**

The chemicals and reagents used were Methanol (Batch No. 6467948, 99%), Sodium carbonate (Batch No.1364110816, 99%), Ferric sulfate (Batch No. 307718, 97%), Potassium iodide (Batch No. P1721, 99%), Bismuth nitrate (Batch No. 24870440, 99%) and silver nitrate (Batch No. 101512, 99%), which were purchased from Alpha Chemika, India. Acetic acid (Batch No.29152100, 99.5%), Aluminum chloride (Batch No.801081, 99%), Sulphuric acid (Batch No.2316395, 98%), Dimethyl sulfoxide (Batch No.276588, 99%), Fehling's reagent, (Batch No.7758987, 97.0%) were obtained from Loba Chemie, India. Hydrochloric acid (Batch No.373647010, 35%) was purchased from (Sisco Research Laboratories, India). Ascorbic acid (Batch No.100468, 99%), Chloroform (Batch No.102447, 99.8%), p-iodonitrotetrazolium chloride (Batch No.4093224, 99.8%) were purchased from Fisher Scientific (United Kingdom). Mueller Hinton Agar and Mueller Hinton Broth were purchased from HiMedia Laboratories (India) whereas DPPH (2, 2-diphenyl-1-picrylhydrazyl, Batch No. 44150, 95%) was obtained from Alfa Aesar (Japan). Analytical-grade chemicals and distilled water were utilized throughout the studies.

**Phytochemical Screening**

**Test for alkaloids (Dragendoff’s test)**

To 1.5 ml of the extract solution 1 ml of Hydrochloric acid (HCl) and 3 drops of Dragendorff’s reagent (Potassium iodide-bismuth nitrate) was added. The formation of orange precipitates or red coloration indicates the presence of alkaloids [1].

**Test for flavonoids (Alkaline reagent test)**

Approximately 1ml of 10% NaOH solution was added to 3 ml of plant leaf extract. The presence of flavonoids can be confirmed by the formation of an intense yellow color [2].

**Test for saponins (Frothing test)**

A 5 ml of distilled water and 0.2 ml plant extract were shaken together. Frothing shows the presence of saponin [3].

**Test for Carbohydrates (Fehling’s test)**

To a few drops of extract, 2 ml of Fehling’s reagent is added. The mixture is shaken well and boils for 5 minutes. Brick red precipitate indicates the presence of sugar [4].

**Test for phenols (Ferric chloride test)**

To 1ml of plant leaf extract 2 ml of a 5% solution of FeCl_3_ was added. The development of black color indicates the presence of phenols [5].

**Test for anthraquinones (Bontrager's test)**

To 0.2 mL of plant leaf extract, 5 mlof chloroform and 5 ml of ammonia solution was added. The presence of bright pink color in the aqueous layer indicated the presence of anthraquinone [6].

**Test for Cardiac Glycosides (Liebermann’s Test)**

To 1ml of plant extract 2.0 ml of acetic acid and 2 ml of chloroform were added and cooled. After that, 1ml of concentrated sulfuric acid was added. The presence of glycosides is indicated by the color green [7].

**LC-MS analysis**

The extract was prepared at a concentration of 1 mg/ml in HPLC grade methanol for LC-MS analysis. A 1 mg of methanol extract was dissolved in 1 ml of fresh HPLC grade methanol and vortexed for 2 min. The mixture was transferred to a 5 ml syringe and filtered through a 0.22 m acrodisc syringe filter into an HPLC vial. LC- MS parameters were Sheath gas flow rate: 35, Aux gas flow rate: 3, Seep gas flow rate: 0, Spray voltage (KV): 3.5, Capillary temperature: 320 ^0^C, S-lens RF level: 50, Aux gas heater temperature: 320 ^0^C. MS conditions: General: Run time 0 to 20 minutes, Default change state: 1.Full MS: resolution: 120, AGC target: 5e4, Maximum, IT: 200 MS, Loop count: 5, Isolation widow: 3.6 m/scan range 100 to 1500 m. Mobile phase A: water and 0.1% formic acid, Mobile phase B: acetonitrile. The results were compared using mzCloud database and Compound discoverer software.

**GC-MS analysis**

The plant extract was prepared at a concentration of 1 mg/ml by dissolving 1 mg of the extract in 1 ml of fresh HPLC grade hexane and vortexed for 2 minutes. The mixture was then transferred to a 5 ml syringe and filtered through a 0.22 m acrodisc syringe filter into an HPLC vial for GC-MS analysis conditions were inlet temperature 250 ^0^C, injection volume 1µl, split ratio 10, Carrier gas helium, column flow 1ml/min. Oven temperature: 40 °C hold for 1 minutes, then 10 °C/min to 160 °C, by 10^o^C/min to 200 °C, by 15 °C/min to 280 ^0^C hold for 3min finally, by 15 °C/min to 290 °C which is hold for 7 min. whereas the MS conditions were Ionization mode: EI , EMV mode: Gain Factor, Gain Factor: 1, Transfer line temperature: 290 °C , Ion Source temp: 230 °C, Quad temp: 150 °C, Solvent delay: 3 minutes and Acquisition mode: Scan, 50–550 amu. The phytochemical constituents were then identified by comparing the results of mass spectrum with inbuilt NIST library database.

**Synthesis of silver nanoparticles**

A 1 ml of the plant extract and a 9 ml of silver nitrate solution was then stirred for 30 minutes in magnetic stirrer at optimized temperature of 45 ^0^C. Following completion, silver nanoparticles were centrifugated and purified for 15 minutes at 12000 rpm using centrifugal ultrafiltration (Hermle Labor Technik, Z 36 HK, Germany) and rinsed with distilled water and ethanol to remove any remaining soluble ions on the particle surface. The nanoparticles were then stored at 4 ^0^C for further characterization. Effects of concentration, reaction time, volume ratio, temperature, and pH on the formation of silver nanoparticles were investigated to optimize the nanoparticles formation .The experiment was carried out by optimizing the concentrations of silver nitrate solution (1 mM, 2 mM, 3 mM, 4 mM, and 5 mM), reaction time (15 min, 30 min, 45 min and 60 min), volume ratio with respect to silver nitrate to plant leaf extract (1:9, 2:8, 3:7, 4:6 and 5:5), temperature of the reaction mixture (25 ^0^C, 35 ^0^C, 45 ^0^C, 55 ^0^C and 65 ^0^C) and effects of pH (pH = 5, pH = 7 pH = 9, pH = 11, and pH =13).

**Antibacterial Activity**

Mueller-Hinton Agar (MHA) was used as the media, and it was prepared according to the manufacturer's instructions, in which 19 g of powder media was mixed with 500 ml of distilled water, sealed in a container, and autoclaved at 121 ^0^C for 15 minutes. The media was then poured into sterile Petri dishes. Following the solidification of the media, 100 µl of the working stock culture was spread with a sterile cotton swab and wells were made in each Petri dish with a stainless-steel cork borer. Then, from a 100 mg/mL stock solution, silver nanoparticle and crude extract of 0.5, 1, 1.5, and 2 mg/ml concentration were prepared. The plant extract was dissolved in DMSO, while the silver nanoparticles were dissolved in distilled water. For the test, DMSO was used as a negative control, Vancomycin (30 μg/disk) and Ciprofloxacin (5 μg/disk) were used as positive control. Vancomycin (30 µg/disk) was utilized for gram positive bacterial strains, and Ciprofloxacin (5 µg/disk) was used for gram negative bacterial strains. After that the petri dishes were incubated at 37 ^0^C for 24 hours. Finally, the inhibition zone of green synthesized silver nanoparticles and plant extract at various concentrations against pathogenic bacteria was measured in millimeters.

A 96-well microplate was used to determine the minimum inhibitory concentration of the plant extract and silver nanoparticles following the procedure by Clinical and Laboratory Standards Institute guidelines [1].

A 2 mg/ml of plant extract and silver nanoparticles was taken and diluted to 1, 0.5, 0.25, 0.125, 0.0625, 0.0315, 0.00156, 0.0078 mg/ml concentration. Each well received 100 µl of broth followed by 100 µl of each sample. Then 20 µL of inoculum were added to each well. The dishes were sealed and incubated at 37 °C for 24 hours. After 24 hours, 40 µl (0.4 mg/mL) p-iodonitrotetrazolium chloride (INT) was added to all wells and incubated for another 24 hours. The broth is used as a bacterial strain growth medium, and the empty wells were used as sterility control in order to ensure that the experiment is carried out without any external contamination.

The minimum bactericidal concentration was effectively determined following the procedure by Hernandes and coworkers [2]. The 96 well plates were incubated for 48 hours at 37 ^0^C, then a volume of 10 µl was removed from the 96 wells where no growth was observed and inoculated onto the surface of agar plates which were prepared by taking 38 g Muller Hinton Agar in 1000 ml distilled water. The plates were then incubated at 37 ^0^C for 48 hours. Both the plant extract and the silver nanoparticles were tested against gram-negative and gram-positive bacterial strains in three replications to determine the minimum concentration capable of inhibiting more than 99.99% of the bacteria present.

**Antioxidant study**

The plant extract and nanoparticle samples were diluted to concentrations of 50, 100, 200, 300, 400, 800, 1000 µg/ml. The standard ascorbic acid was diluted to concentrations of 2.5, 5, 10, 15, 20, 25, 30, 40, 80 and 100 µg/ml. The control and the samples (1 ml each) were reacted with DPPH (5 ml) each in falcon tubes for 30 minutes in the dark.

Methanol was used as a blank and the absorbance of the sample was recorded using UV spectrometer (Sunny Hengping Scientific, UV756, China) at 517 nm. The mean of each triplicate was taken for the analysis. The DPPH reduction percentage is calculated as follows:

$\frac{Ao-As}{Ao}\times100$

A_o_ and A_s_ are the blank and sample absorbance, respectively. The IC50 values can be calculated from the DPPH reduction percentages and referred to the concentration of the sample (in µg/ml) required to reduce 50% of the DPPH present in the assay.

**Results and discussion**


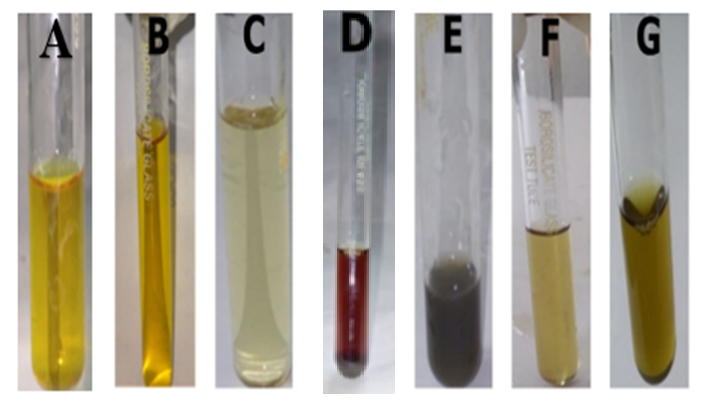


**Figure S1**. The color change observed when leaf extract of *Verbascum sinaiticum* were tested for the presence or absence of (A) Alkaloids, (B) Flavonoids, (C) Saponins, (D) Carbohydrate, (E) Phenols, (F) Anthraquinone, (G) Cardiac glycosides.

**Table S1**. **Identified compounds in methanol extracts of *Verbascum* *sinaiticum* in LC-MS**

| **Compound Name** | **Molecular Formula** | | **Retention time [min]** | | **Class of compound** |
| --- | --- | --- | --- | --- | --- |
| Verbascoside A | C_31_ H_40_ O_16_ | | | 6 | Phenylpropanoids |
| Scropheanoside I | C_31_ H_40_ O_16_ | | | 6.319 | Polyphenols |
| Picroside II | C_23_ H_28_ O_13_ | | | 4.24 | Tannin |
| Phlinoside A | C_35_ H_46_ O_20_ | | | 4.149 | Phenylpropanoids |
| N-Acetyl-L-leucine | C_8_ H_15_ NO_3_ | | | 4.297 | alpha amino acids. |
| Methyl succinic acid | C_5_ H_8_ O_4_ | | | 2.659 | Acrylic acid esters. |
| L-Glutathione oxidized | C_20_H_32_N_6_O_12_S_2_ | | | 1.367 | Disulfide |
| Harpagoside | C_24_ H_30_O_11_ | | | 6.657 | Glycoside |
| Guanosine | C_10_ H_13_ N_5_O_5_ | | | 1.599 | Purine nucleosides |
| Glycoursodeoxycholic acid | C_26_ H_43_ NO5 | | | 9.447 | Acyl glycine |
| Glutaric acid | C_5_H_8_O_4_ | | | 2.211 | Dicarboxylic acids |
| Gluconic acid | C_6_ H_12_O_7_ | | | 0.931 | Carboxylic acid |
| Geniposidic acid | C_16_H_22_O_10_ | | | 2.722 | Iridoid glucoside |
| Fumaric acid | C_4_H_4_O_4_ | | | 1.036 | Dicarboxylic acids |
| DL-Arginine | C_6_H_14_N_4_O_2_ | | | 0.868 | Alpha-amino acid |
| D-Raffinose | | C_18_H_32_O_16_ | | 0.948 | Trisaccharide |
| D- (-)-Lyxose | | C_5_H_10_O_5_ | | 1.039 | Carbohydrates |
| D- (-)-Glutamine | | C_5_H_10_N_2_O_3_ | | 0.903 | d-alpha-amino acids. |
| D- (-)-Fructose | | C_6_ H_12_O_6_ | | 1.001 | Monosaccharide |
| D- (+)-Mannose | | C_6_H_12_O_6_ | | 0.912 | Carbohydrate |
| D- (+)-Galactose | | C_6_ H_12_O_6_ | | 0.904 | Hexoses |
| Cynaroside | | C_21_ H_20_ O_11_ | | 5.034 | beta-D-glucoside |
| Corchorifatty acid F | | C_18_ H_32_ O_5_ | | 7.51 | Lineolic acids |
| Citric acid | | C_6_ H_8_ O_7_ | | 1.365 | Tricarboxylic acid |
| 4-Coumaric acid | | C_9_ H_8_ O_3_ | | 3.681 | Hydroxycinnamic acids. |
| 4-(4-Hydroxyphenyl)-2-butanyl6-O-[(4ξ)-α-L-threo-pentofuranosyl]-β-D-glucopyranoside | | C_21_ H_32_ O_11_ | | 6.081 | Benzenoids |
| 3-Hydroxy-3-(methoxycarbonyl)pentane dioic acid | | C_7_ H_10_ O_7_ | | 2.051 | Carboxylic acid |
| 3-[2-(1,3-Benzodioxol-5-yl)-7-methoxy-1benzofuran-5-yl]-3-hydroxypropyl hexopyranoside | | C_25_ H_28_ O_11_ | | 0.92 | Glycoside |
| 3,4,5-trihydroxycyclohex-1-ene-1-carboxylic acid | | C_7_ H_10_ O_5_ | | 1.183 | Carboxylic acid |
| 2-Hydroxy-2-methyl-3-buten-1-ylbeta-D-glucopyranoside | | C_11_ H_20_ O_7_ | | 1.876 | Glycoside |
| 2-Anisic acid | | C_8_ H_8_ O_3_ | | 2.069 | Benzoic acid |
| 2-[3,8-Dihydroxy-8-(hydroxymethyl)-3-methyl-2-oxodecahydro-5-azulenyl]-2-propanyl hexopyranoside | | C_21_ H_36_ O_10_ | | 6.825 | Glycoside |
| 2-(hydroxymethyl)-6-[(E)-4-(1,2,4-trihydroxy-2,6,6-trimethylcyclohexyl)but-3-en-2-yl] oxyoxane-3,4,5-triol | | C_19_ H_34_ O_9_ | | 3.542 | Carboxylic acid. |
| 2-(6-Hydroxyhexyl)-3-methylene succinic acid | | C_11_ H_18_ O_5_ | | 6.324 | Dicarboxylic acid |
| 2-(4-Methyl-3-cyclohexen-1-yl)-2-propanyl6-O-(6-deoxy-α-L-mannopyranosyl)-β-D-glucopyranoside | | C_22_ H_38_ O_10_ | | 6.648 | Glycoside |
| 2-(4-Hydroxyphenyl)ethyl6-O-[(2R,3R,4R)-3,4-dihydroxy 4(hydroxymethyl)tetrahydro-2-furanyl]-beta-D-glucopyranoside | | C_19_ H_28_ O_11_ | | 3.429 | Glycosides |
| 13(S)-HOTrE | | C_18_ H_30_ O_3_ | | 11.315 | linolenic acid. |
| (±)9-HpODE | | C_18_ H_32_ O_4_ | | 9.876 | linolenic acids |
| (±)-Abscisic acid | | C_15_ H_20_ O_4_ | | 6.673 | Terpenoid |
| (2S,3R,4S,5S,6R)-2-({4a,5,7-trihydroxy-7-methyl-1H,4aH,5H,6H,7H,7aH-cyclopenta[c]pyran-1-yl}oxy)-6-(hydroxymethyl)oxane-3,4,5-triol | | C_15_ H_24_ O_10_ | | 2.627 | Carboxylic acid |
| (2E)-3-Phenyl-2-propen-1-yl6-O-beta-D-arabinofuranosyl-beta-D-glucopyranoside | | C_20_ H_28_ O_10_ | | 4.028 | Glycoside |
| (15Z)-9,12,13-Trihydroxy-15-Octadecenoic acid | | C_18_ H_34_ O_5_ | | 7.938 | Fatty acid |
|  | |  | |  |  |

**Figure S2**. Total ion chromatogram of Verbascum sinaiticum from GC-MS.

List of compounds in decreasing order of volatility from top to bottom is shown in Table S1. Nonadecane (C_19_H_40_) was found to be the most volatile compound, with an 8.22 retention time and a percentage area of 0.45, while Stigmasterol (C_29_H_48_O) was found to be the least volatile, with a 31.06 retention time and a percentage area of 0.68. The highest percentage of compound found in the extract were Hentriacontane, Tetratriacontane, Octacosane, 2, 4-Di-tert-buthylphenol and 9, 12, 15-0ctadecatrienoic acid methyl ester, (ZZZ). These compounds are reported to have antibacterial, antifungal, anti-inflammatory and anticancer activities [3].

**Table S2.** **Identified compounds in leaf extracts of *Verbascum sinaiticum* in GC-MS**

| **Compound Name** | **Formula** | | **Retention time** | | **%Area** | | **Class of compounds** |
| --- | --- | --- | --- | --- | --- | --- | --- |
| Nonadecane | C_19_H_40_ | 8.22 | | | 0.45 | Alkane | |
| Hexadecane, 2,6.11, 15-tetramethyl | C_20_H_42_ | 8.52 | | | 1 .20 | Alkane | |
| 2,4-Di-tert-buthylphenol | C_14_H_22_O | 9.93 | | 4.90 | | Phenylpropanes | |
| Hexadecane | C_16_H_34_ | 12.83 | | 0.27 | | Alkane | |
| Sedoheptulose, 2,3:4,5-dimethylene | C_9_H_14_O_7_ | 13.35 | | 0.56 | | Ketose | |
| 3,5-Di-tert-butylphenol | C_14_H_22_O | 14.08 | | 0.52 | | Phenols | |
| Heneicosane | C_21_H_44_ | 14.82 | | 0.71 | | Alkane | |
| Hexadecane, 2,6,11,15-tetramethyl | C_20_H_42_ | 14.89 | | 1.30 | | Alkane | |
| Phytane | C_20_H_42_ | 14.98 | | 0.25 | | Alkane | |
| 1-Decanol, 2-hexyl | C_16_H_34_O | 16.84 | | 0.18 | | Alcohol | |
| Phyto1 | C_20_H_40_O | 17.06 | | 0.97 | | Fatty acid | |
| Hexadecanoic acid, methyl ester | C_17_H_34_O_2_ | 18.11 | | 2.43 | | Ester | |
| 9, 12, 15-0ctadecatrienoic acid, methyl ester, (ZZZ) | C_19_H_32_O_2_ | 19.69 | | 3.50 | | Ester | |
| Tetradecane, 2,6, 10-trimethyl | C_17_H_36_ | 19.81 | | 1.62 | | Sesquiterpenoids | |
| Pentacosane  Heptadecane, 2.6, 10, 15-tetramethyl | C_25_H_52_  C_21_ H_44_ | 20.05  20.13 | | 0.26  0.65 | | Alkane  Alkane | |
| Batilol | C_21_H_44_0_3_ | 21.25 | | 0.91 | | Alkylglycerol | |
| Nonane, 2-bromo-5-ethyl | C_11_H_23_Br | 21.30 | | 1.10 | | Alkane | |
| 7-Methyl-Z-tetradecen-l-ol acetate | C_17_H_32_O_2_ | 21.63 | | 0.20 | | Methyl ester | |
| 1 ,1,3,6-tetramethyl-2-(3,6, 10, 13, 14-pentamethyl-3-ethyl-pentadecyl) cyclohexane | C_32_H_64_ | 22.78 | | 0.29 | | Alkane | |
| Heptacosane | C_27_H_56_ | 23.65 | | 1.91 | | Alkane | |
| Octatriacontyl pentafluoroproionate | C_41_H_77_F_5_ O_2_ | 24.52 | | 0.51 | | Ester | |
| alpha -Tocospiro A | C_29_H_50_O_4_ | 25.06 | | 0.37 | | Terpenoid | |
| 1±-Tocospiro B | C_29_H5004 | 25.25 | | 1 .26 | |  | |
| Octacosane | C_28_H_5_S | 25.48 | | 11.16 | | Glycoside esters | |
| Nonacosane, 3-methyl | C_30_H_62_ | 26.17 | | 2.06 | | Alkane | |
| Nonacosane | C_29_H_60_ | 26.43 | | 2.70 | | Alkane | |
| Tetratetracontane | C_44_H_90_ | 27.13 | | 2.33 | | Alkane | |
| Hentriacontane | C_31_H_64_ | 27.62 | | 30.81 | | Alkane | |
| Triacontane. 1-bromo | C_30_H_61_Br | 28.56 | | 2.86 | | Alkane | |
| Hexatriacontane | C_36_H_74_ | 28.94 | | 3.87 | | Alkane | |
| Tetratriacontane | C_34_H_70_ | 30.62 | | 13.73 | | Alkane | |
| Stigmasterol | C_29_H_48_O | 31.06 | | 1 .68 | | Sterols | |


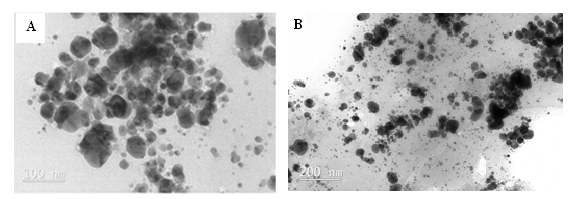


**Figure S3**. TEM images in (A) 100 nm and (B) 200 nm scale showing spherical shape of the synthesized silver nanoparticles from *Verbascum sinaiticum* leaf extract.


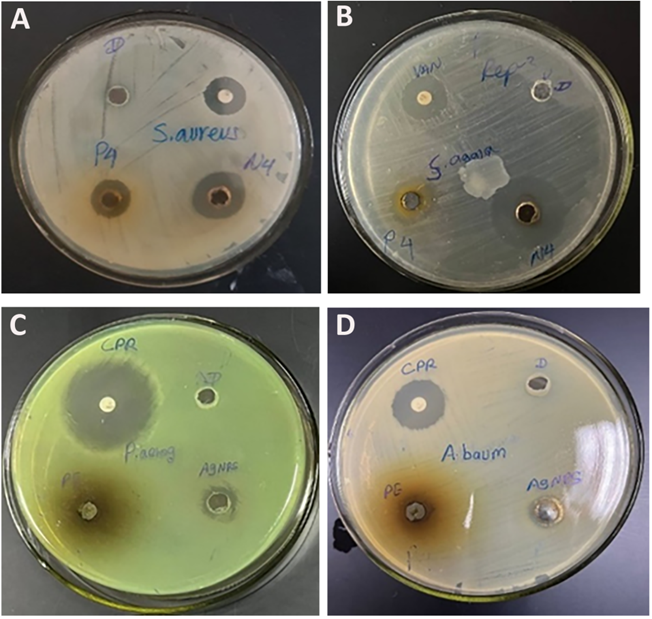


**Figure S4.** Agar well diffusion result of plant extract and silver nanoparticles against four bacterial strains (A) *Staphylococcus aureus*, (B) *Streptococcus agalactiae*, (C) *Pseudomonas aeruginosa* and (D) *Acinetobacter baumannii* bacterial strain tested P = Plant extract, N = Nanoparticles and D = DMSO in all A, B, C and D plates.


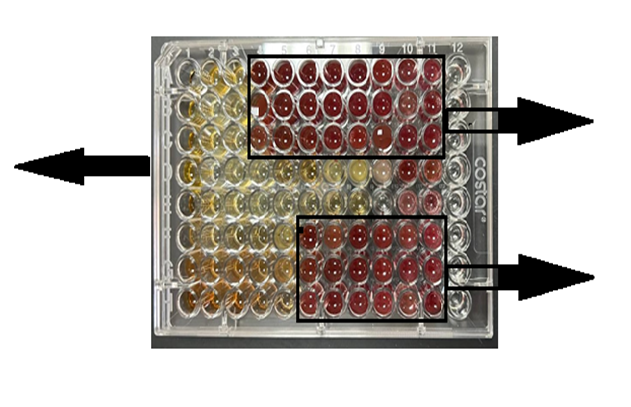


1 A_1_--3C_3_ Shows inhibition of bacterial growth by the plant extract

4 A_1_- 11C_11_-Shows color change occurring Due to the inability of plant extract to inhibit the bacterial growth

D_1_-D9 -Amoxicillin

D_10_-DMSO+broth

D_11_-Growth control

D_12_-Sterility Control

1F_1_-5H_5-_Shows inhibition of bacterial growth by silver nanoparticles

6F_6_-11H_11-_Color change occurs due to the inability of silver nanoparticles to inhibit bacterial growth.

**Figure S5.** MIC determination of the plant extracts and synthesized silver nanoparticles using 96-well plate.

**
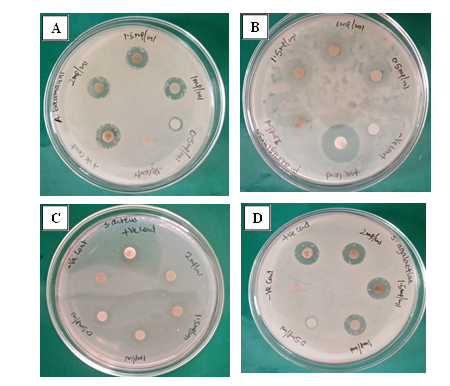
**

**Figure S6.** Antibacterial activity of silver nitrate at 0.5 mg/ml, 1 mg/ml, 1.5 mg/ml and 2 mg/ml against A) *A .baumann* B) *P. aureginosa,* C) *S.aureus,* D) *S. agalactiae*.

**
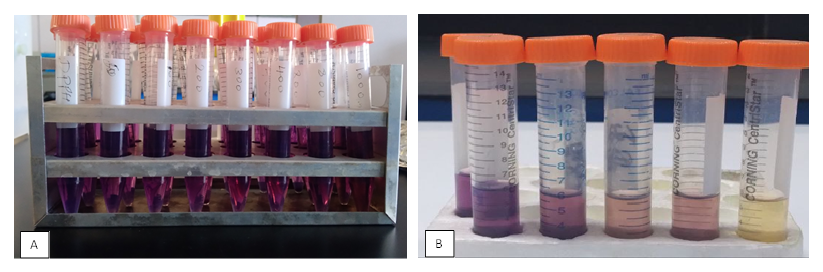
**

**Figure S7. DPPH assay antioxidant tests of a) silver nitrate and b) the plant extract.**

As shown from the pictures above(Figure S7(A)) no reaction with silver nitrate because reaction usually is followed by color change to yellow appearance rather than maintaining the DPPH purple color. However, the color change was observed for the plant extract Figure S7(B). Because of reduction, the color of the DPPH was changed from purple to yellow.

In addition, silver nitrate is not an antioxidant because it does not possess the essential characteristics and mechanisms of action typically associated with antioxidants. An antioxidant is a substance that helps protect cells from damage caused by free radicals. They work by neutralizing free radicals through various mechanisms, such as donating an electron to stabilize the free radical or scavenging reactive oxygen species. These actions help prevent cellular damage and promote overall health. However, silver nitrate does not possess these antioxidant properties.

**References**

1. Zakaria, Z. A., Balan, T., Mamat, S. S., Mohtarrudin, N., Kek, T. L., & Salleh, M. Z. (2015). Mechanisms of gastroprotection of methanol extract of Melastoma malabathricum leaves. BMC complementary and alternative medicine, 15(1), 1-15.
2. Patil, M. S., Chittam, K. P., & Patil, S. B. (2021). Phytochemical screening of Sesbania grandiflora L. bark. Journal of Pharmacognosy and Phytochemistry, 10(4), 153-158.
3. Omokpariola, D., Precious-Egere, S., Omokpariola, P., & Okechukwu, V. (2021). Phytochemical and Anti-Microbial Analysis of Metabolites in seeds of Moringa oleifera grown in Nigeria. Progress in Chemical and Biochemical Research, 4(3), 268-277.
4. Parihar, H. R., Gaur, A., Singh, K., Sharma, P., & Panwar, A. (2022). Qualitative phytochemical screening of various extracts of Cucurbita maxima seeds.
5. Kardong, D., Upadhyaya, S., & Saikia, L. (2013). Screening of phytochemicals, antioxidant and antibacterial activity of crude extract of Pteridium aquilinum Kuhn. Journal of pharmacy research, 6(1), 179-182.
6. Melkamu, W. W., & Bitew, L. T. (2021). Green synthesis of silver nanoparticles using Hagenia abyssinica (Bruce) JF Gmel plant leaf extract and their antibacterial and anti-oxidant activities. Heliyon, 7(11).
7. Mathew, B. B., Jatawa, S. K., & Tiwari, A. (2012). Phytochemical analysis of Citrus limonum pulp and peel. Int J Pharm Pharm Sci, 4(2), 369-71.
8. C. CLSI, Performance standards for antimicrobial susceptibility testing. Clinical Lab Standards Institute, 2016. **35**(3): p. 16-38
9. Hernandes C., Flash microbiocide: A rapid and economic method for determination of MBC and MFC. Am. J. Plant Sci, 2013. **4**: p. 850-852
10. N.U. Rehman, Carbonic Anhydrase-II, α-Glucosidase, and Chemical Composition of Essential Oils from Stem and Leaves of Zygophyllum qatarense. Journal of Essential Oil Bearing Plants, 2022. **25**(4): p. 835-843.
